# The GPER Agonist LNS8801 Induces Mitotic Arrest and Apoptosis in Uveal Melanoma Cells

**DOI:** 10.1158/2767-9764.CRC-22-0399

**Published:** 2023-04-05

**Authors:** Grazia Ambrosini, Christopher A. Natale, Elgilda Musi, Tina Garyantes, Gary K. Schwartz

**Affiliations:** 1 The Herbert Irving Comprehensive Cancer Center, Columbia University Medical Center, New York, New York.; 2 Linnaeus Therapeutics, Haddonfield, New Jersey.; 3 Division of Hematology/Oncology, Columbia University Medical Center, New York, New York.

## Abstract

Uveal melanoma is the most common primary intraocular malignancy in adults and has a high incidence of metastatic disease. Current treatments have shown limited clinical activity in patients with uveal melanoma with metastasis and there is an urgent need for new effective therapies. Recent findings have shown that women with uveal melanoma have better survival rates than men. The G protein–coupled estrogen receptor-1 (GPER) has distinct functions from those of the classic estrogen receptors ERα/β and its activation by specific agonists has tumor-suppressive roles in several cancers. However, the role of GPER had not previously been investigated in uveal melanoma. We demonstrated that downregulation of GPER in uveal melanoma cells decreased expression of p53 and stimulated cell growth. In contrast, the clinical GPER agonist, LNS8801, upregulated p53 and p21, induced melanocytic differentiation markers, inhibited cell proliferation and cell migration, and induced apoptosis. Furthermore, LNS8801 treatment arrested the cells in G_2_–M-phase of the cell cycle with concomitant activation of mitotic markers and disruption of the mitotic spindle apparatus. LNS8801 significantly inhibited tumor growth of uveal melanoma xenografts *in vivo*, suggesting that GPER agonists may be a novel treatment for uveal melanoma.

**Significance:**

Current treatments against metastatic uveal melanoma have shown limited clinical activity and there is an urgent need for effective therapies. Here, we demonstrate that the GPER agonist LNS8801 induced both GPER-dependent and GPER-independent effects and elicited potent anticancer activities *in vitro* and *in vivo*. Our results complement and support the ongoing clinical trial of LNS8801 in advanced uveal melanoma.

## Introduction

Uveal melanoma is the most common intraocular malignant tumor in adults (1). Despite effective therapy for the primary tumor with surgery and radiotherapy, 50% of the patients eventually develop metastases, mostly into the liver (2). Poor prognosis is associated with monosomy 3, 6q loss and isochromosome 8q, and classified as class I and II by gene expression (3). The median survival rate for these patients is 12–15 months (4). Identifying new effective therapies to arrest local and systemic disease is critical. Several reports have shown gender differences in the clinical presentation and prognosis of patients with uveal melanoma, with higher incidence and metastasis rates in men compared with women (5–7). Previous studies have determined that estrogen, especially during pregnancy, acts directly on primary melanocytes to increase both pigment production and melanocyte differentiation through G protein–coupled estrogen receptor (GPER; ref. 8). Furthermore, selective GPER activation in primary melanocytes and melanoma cells induced long-term changes that maintained a more differentiated cell state that is less tumorigenic (9). GPER, also called GPR30, is expressed in a variety of tissues including the nervous, reproductive, digestive, and muscle apparatus (10, 11) and it plays a role in the regulation of numerous cell functions in health and disease (11, 12). Estrogen can directly bind to and agonize GPER which leads to regulation of multiple downstream signals such as ERK1/2, PI3K/Akt, and Hippo/YAP/TAZ pathway (13). GPER is also involved in melanogenesis via cAMP-protein kinase by regulating microphthalmia-associated transcription factor (MITF) and tyrosinase (TYR; ref. 14). To distinguish GPER-mediated estrogen action from that of the classic ERα and ERβ, selective GPER agonists and antagonists have been developed (15, 16). Activation of GPER with the preclinical, nonsteroidal GPER agonist G-1, has tumor-suppressive effects in several cancers (17–19). In cutaneous melanoma, activation of GPER inhibited cell proliferation and improved response to immune checkpoint blockade (9). In ovarian cancer and glioblastoma cells, G-1 inhibited cell proliferation, arrested the cells in G_2_–M-phase of the cell cycle, and blocked tubulin polymerization. However, these effects were independent of GPER (19, 20).

LNS8801 is an orally available, small-molecule GPER agonist, currently in clinical trials for solid tumors including uveal melanoma (NCT04130516). Here, we demonstrate that the GPER agonist LNS8801 induces differentiation markers and inhibits uveal melanoma cell proliferation and migration. Furthermore, LNS8801 induces the formation of defective mitotic spindles and an arrest of the cell cycle in G_2_–M. LNS8801 has potent antitumor effects in uveal melanoma xenografts, and it may represent a promising therapy against this aggressive disease.

## Materials and Methods

### Cell Lines and Reagents

Omm1.3 and Omm1 cell lines (RRID:CVCL 6939) were kindly provided by Dr B. Bastian, University of California, San Francisco, CA. The cell line 92.1 was provided by Dr W. Harbour, UT Southwestern Medical Center, Dallas, TX. MP41 cells (ATCC # CRL-3297) were purchased from ATCC. Uveal melanoma cells were validated by sequencing of the GNAQ/11 mutations and cultured in RPMI medium supplemented with 10% FBS, 100 units/mL penicillin, and 100 μg/mL streptomycin and maintained at 37°C in 5% CO_2._ The cells were tested for *Mycoplasma* using the MycoAlert *Mycoplasma* Detection Kit (Lonza). The cells were grown for no more than 20 passages and discarded. LNS8801 was provided by Linnaeus Therapeutics. G-36 was purchased from Cayman Chemical Company.

### siRNA Transfections

siRNAs against GPER were purchased from Santa Cruz Biotechnology (GPER-a) and Thermo Fisher Scientific (GPER-b) and transfected into the cells using Lipofectamine RNAiMax reagent (Thermo Fisher Scientific), according to package instructions. The cells were counted after 3 and 6 days from transfection in triplicate samples using a Nexcelom Bioscience cell counter. Experiments were repeated three times and reported as mean ± SD.

### Immunoblotting

The cells were lysed in RIPA buffer (Cell Signaling Technology) supplemented with protease inhibitor cocktail tablets (Roche Diagnostics). Total protein concentration of the lysates was measured by BCA assay (Bio-Rad), and equal amounts of protein were loaded on 4%–12% PAGE gels (Thermo Fisher Scientific). Polyvinylidene difluoride membranes were blocked with 5% nonfat dried milk in TBS buffer and probed with antibody for GPER, glycoprotein 100 (gp100; Abcam), p53, TYR (Santa Cruz Biotechnology), p21, caspase 3, MITF, GAPDH (Cell Signaling Technology).

### Transwell Migration Assay

The cell migration assays were performed with 5 × 10^4^ Omm1.3 and MP41 cells seeded in 500 μL serum-free RPMI medium on Cytoselect cell migration chambers (Cell Biolabs). The cells were treated with vehicle (DMSO) or 100 nmol/L LNS8801 for 24 hours. The migrated cells were fixed in methanol and stained with 1% Toluidine Blue. Images of stained cells were taken with a phase contrast microscope.

### Cell Viability Assays

Cell viability was measured after 3 days of treatments in 96-well plates using the Cell Counting Kit 8 from Dojindo Molecular Technologies, and expressed as a percentage of untreated cells. Apoptosis was measured using Apo-ONE Homogeneous Caspase-3/7 Assay (Promega) following the manufacturer’ instructions. Fluorescence signals were analyzed on Varioskan LUX microplate reader (Thermo Fisher Scientific).

### Melanin Assay

Cells (1 × 10^5^) were seeded on 6-well plates, then treated with DMSO or LNS8801 for 4 days. The cells were then trypsinized and counted. The resulting cell pellet was solubilized in 1 mol/L NaOH and boiled. The optical density of the resulting solution was read at 450 nm using an EMax microplate reader. The absorbance was normalized to the number of cells in each sample.

### Flow Cytometry

Cells were fixed in 70% ice-cold ethanol before staining with propidium iodide (50 μg/mL) containing RNase (5 μg/mL) for the measurement of DNA content. Mitotic population was measured by labeling with phospho-MPM-2 mAb (Millipore), followed by Alexa Flour 488 secondary antibody (Thermo Fisher Scientific). Samples were analyzed on a LSR II flow cytometer (Becton Dickinson) for cell-cycle distribution and mitotic index (fraction of cells positive for phospho-MPM2) using the BD FACSDiva software. Analysis of data was completed using FCS 7 Express software.

### Fluorescent Immunocytochemistry

Cells were cultured on chamber slides and treated with LNS8801 for 24 hours. Cells were fixed in 4% formaldehyde, permeabilized in 0.05% tween, blocked with 2% FBS, and incubated with an anti-α-tubulin FITC antibody (Sigma-Aldrich) for 1 hour. Cells were washed with PBS and stained with 4,6-diamino-2-phenylindole (DAPI). Images were acquired on Nikon A1 confocal microscope at 60× magnification and visualized with Fiji ImageJ.

### Xenograft Studies

Athymic nu/nu mice were purchased from Taconic and used when they were 8 weeks old. 92.1 cells (10 × 10^6^) were inoculated subcutaneously into the right flanks of the mice. When the tumors reached an average of 400 mm^3^ volume, the mice (9/group) were administered with vehicle or 1 mg/kg of LNS8801. The treatment was administered daily by oral gavage for 5 weeks. Tumor size was measured twice a week. Experiments were carried out under an Institutional Animal Care and Use Committee–approved protocol, and Institutional guidelines for the proper and humane use of animals were followed. Statistical significance was determined by two-way Student *t* test and a longitudinal mixed-effects model (21).

### Data Availability Statement

The data generated in this study are available upon request from the corresponding author.

## Results

### LNS8801 Inhibits Cell Proliferation and Induces Apoptosis in Uveal Melanoma Cells

**FIGURE 1:**
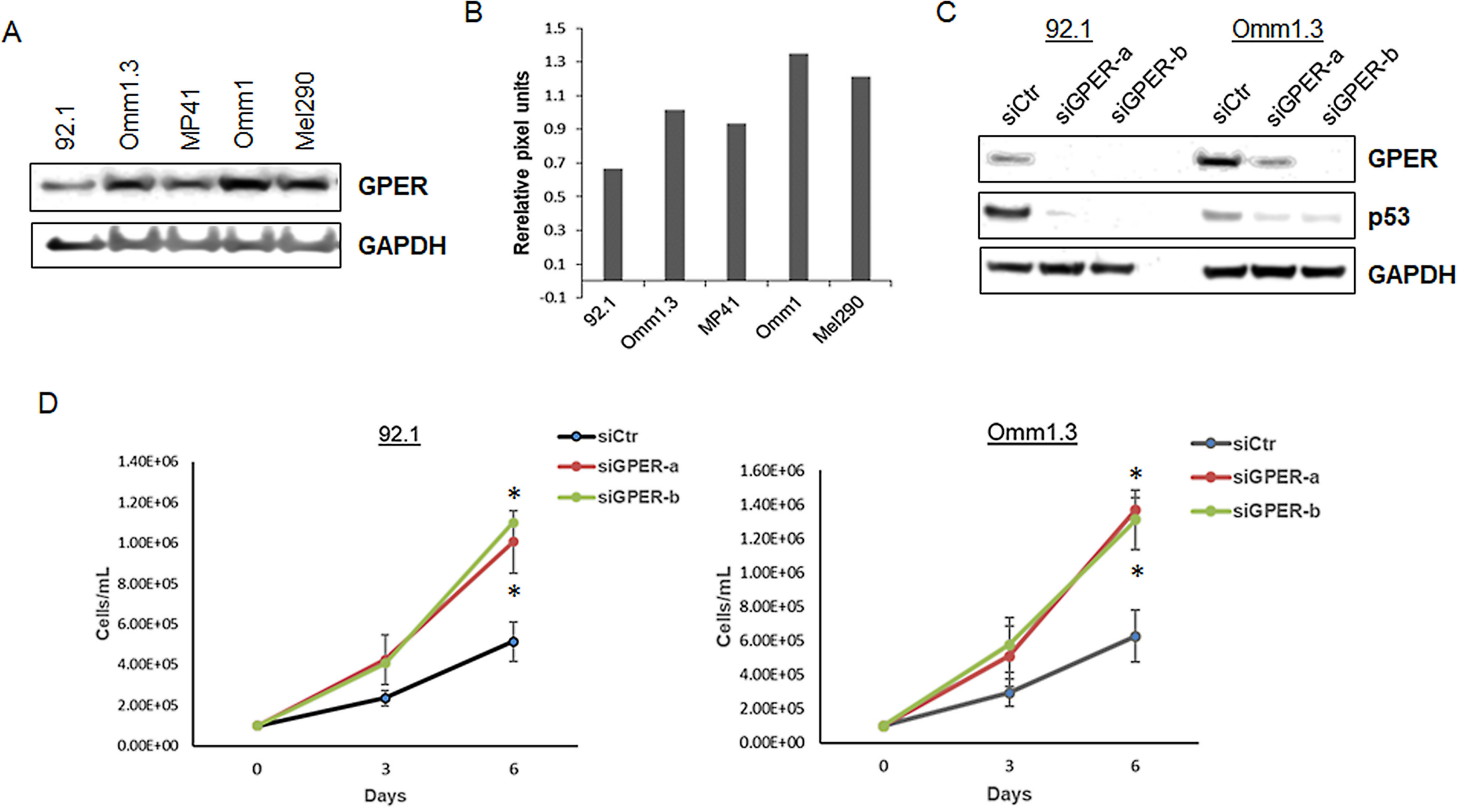
GPER downregulation induces uveal melanoma cell growth. **A,** Expression of GPER in five uveal melanoma cell lines by immunoblotting and densitometry analysis. **B,** GAPDH was the loading control. **C,** Immunoblotting of 92.1 and Omm1.3 cells after transfection with control (siCTR) and two GPER-specific siRNA (siGPER-a and siGPER-b), showing the expression of GPER and p53. **D,** Cell growth of both cell lines over time after GPER depletion compared to control siRNA-transfected cells. Mean ± SD of three independent experiments. *, *P* < 0.05.

We analyzed the expression of GPER in five uveal melanoma cell lines and found that GPER was expressed in all cell lines at comparable levels by immunoblotting and densitometry analysis (Fig. 1A and B). GPER was described as a tumor suppressor gene in ovarian and breast cancer (22, 23) and its activation was shown to regulate the expression of p53 (24), another tumor suppressor and cell-cycle regulator. To examine the effect of GPER knockdown in uveal melanoma, 92.1 and Omm1.3 cells were transfected with control siRNA and two GPER-specific siRNA (siGPER-a and siGPER-b). GPER silencing led to the downregulation of p53 (Fig. 1C). The cells were counted at days 0, 3, and 6 after siRNA transfection to measure cell growth. GPER silencing resulted in a significant increase in cell number over time in both cell lines compared with control cells (Fig. 1D), suggesting that GPER regulates p53 expression and cell growth.

**FIGURE 2:**
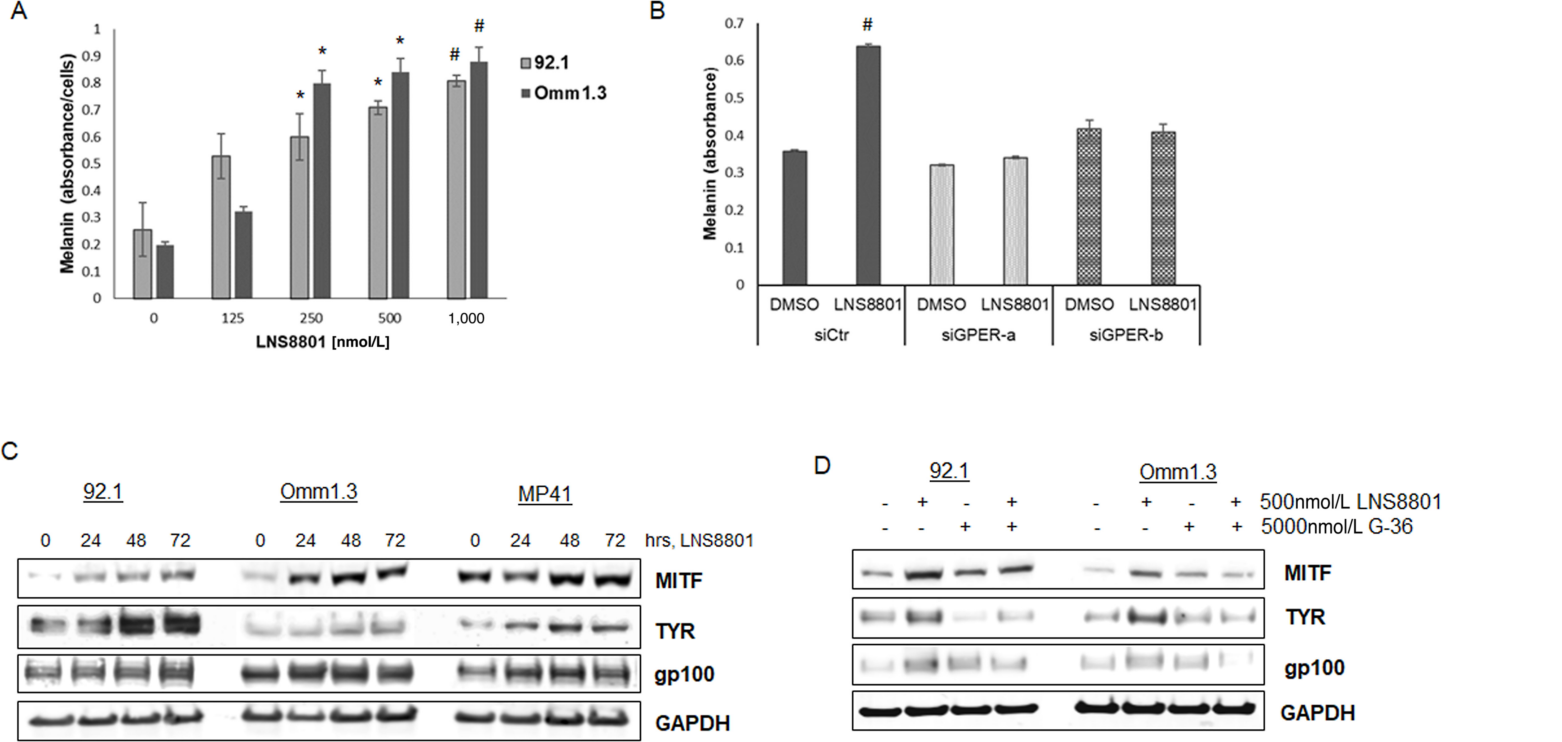
The GPER agonist LNS8801 induces cell differentiation markers. **A,** Melanin production was quantitated in 92.1 and Omm1.3 cell after treatment with increasing doses of LNS8801 for 4 days. A representative experiment done in triplicates is shown *, *P* < 0.05; ^#^, *P* < 0.001. **B**, GPER was downregulated in 92.1 cells and analyzed for melanin production after treatment with 500 nmol/L LNS8801. Bars are triplicate means ± SD. ^#^, *P* < 0.001. **C,** Uveal melanoma cell lines (92.1, Omm1.3 and MP41) were treated with 500 nmol/L LNS8801 for the indicated times and analyzed by Western blotting using antibodies for MITF, TYR, and gp100. **D,** Uveal melanoma cells were pretreated with 5,000 nmol/L G-36 before treatment with 500 nmol/L LNS8801 for 48 hours and analyzed by Western blotting for the indicated proteins.

GPER is involved in estrogen-mediated melanin synthesis (14) and the preclinical agonist, G-1, was shown to induce both pigment production (8, 9) and melanocyte differentiation (14). Similarly, we found that treatment with LNS8801 for 4 days induced a substantial dose-dependent increase of melanin in uveal melanoma cells (Fig. 2A). This effect was abrogated by GPER downregulation by siRNA (siGPER-a and siGPER-b; Fig. 2B), confirming that the effect on melanin by LNS8801 was dependent of GPER expression. In addition, after exposure to LNS8801, uveal melanoma cell lines showed a time-dependent increase of MITF, which is known as the master regulator of melanocyte differentiation (25), and TYR a key enzyme in melanin synthesis (ref. 26; Fig. 2C). LNS8801 also induced gp100, a protein involved in melanosome maturation (27). As a control, we used the specific GPER antagonist G-36 (16) to pretreat the cells at 10-fold higher concentration (5,000 nmol/L) for 4 hours before treating the cells with 500 nmol/L LNS8801 for 48 hours. G-36 mitigated the induction of MITF, TYR, and gp100 (Fig. 2D), further confirming these GPER-dependent effects.

**FIGURE 3:**
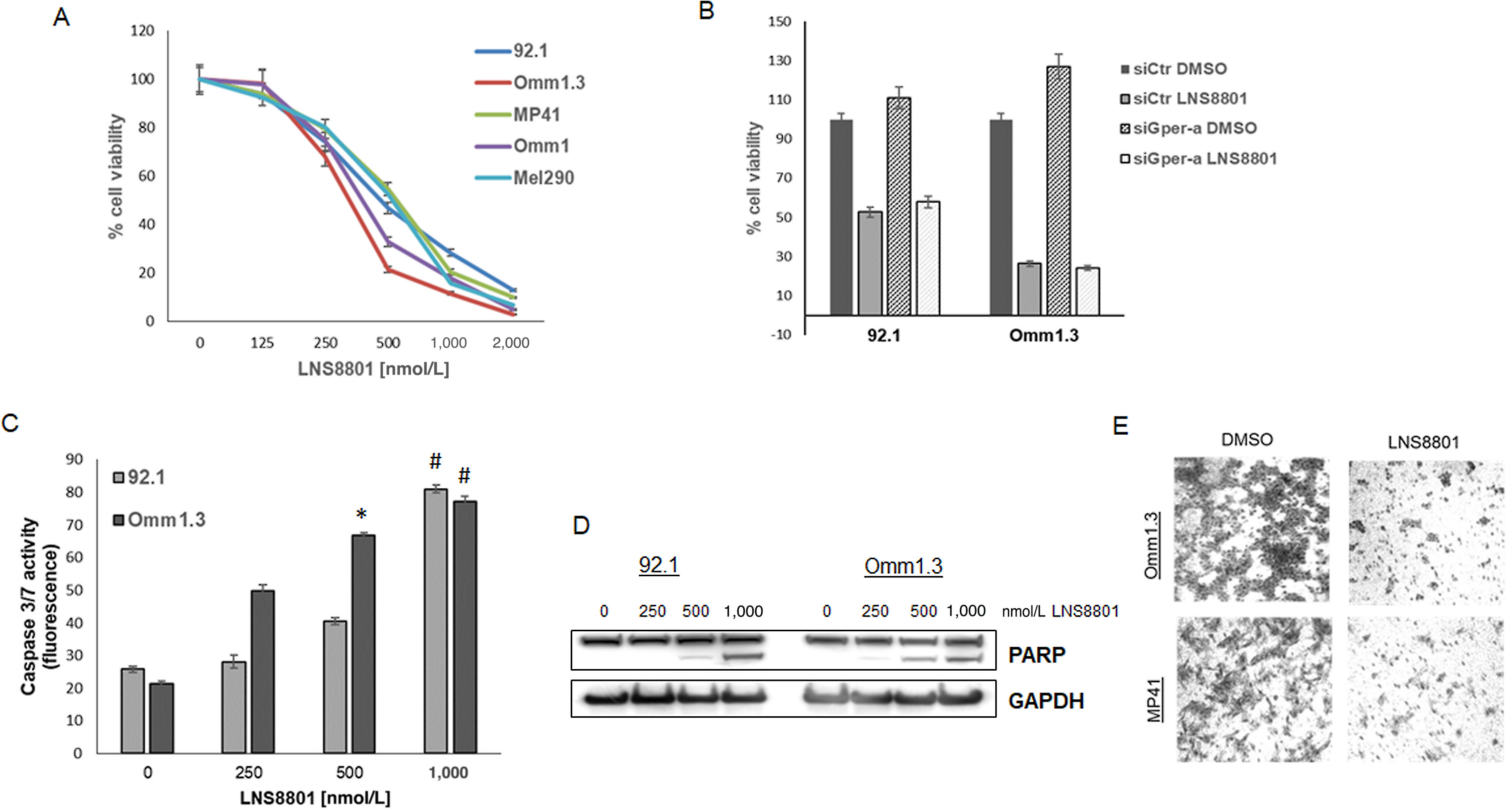
LNS8801 inhibits uveal melanoma cell viability and induces apoptosis. **A,** Uveal melanoma cells were exposed to 2-fold serial dilutions from 2,000 to 100 nmol/L of LNS8801 in sextuplicate for 4 days, and viability was normalized to DMSO-treated cells. Data points are mean ± SD. **B,** Uveal melanoma cells were transfected with siRNA Control and siGPER-a, then treated with LNS8801 for 72 hours. A representative experiment in triplicates is shown. Mean ± SD. **C,** The cell lines 92.1 and Omm1.3 were exposed to increasing concentrations of LNS8801 (0–1,000 nmol/L) for 24 hours, then treated with a profluorescent DEVD peptide substrate and caspase 3 and 7 activity. Assays were done three times in triplicates. Mean ± SD; *, *P* < 0.05; ^#^, *P* < 0.001. **D**, 92.1 and Omm1.3 cells were analyzed by immunoblotting for cleaved PARP after 24 hours treatments with increasing doses of LNS8801. **E,** LNS8801 inhibits cell invasion at 100 nmol/L concentration after 24 hours. The experiment was repeated three times.

Next, we analyzed the effect of LNS8801 on cell growth. All uveal melanoma cell lines treated with LNS8801 showed a dose-dependent decrease in cell viability, with an IC_50_ of 250–500 nmol/L (Fig. 3A). However, downregulation of GPER by siRNA did not counteract the effect of LNS8801 (Fig. 3B), suggesting that the decrease in cell viability was independent of GPER. At doses of 250–1,000 nmol/L, LNS8801 induced apoptosis, as detected by caspase 3/7 activation (Fig. 3C) and induction of PARP cleavage (Fig. 3D) in both 92.1 and Omm1.3 cells. We elected to test the effect of LNS8801 in migration assays in two uveal melanoma metastatic cell lines, MP41 and Omm1.3, using a drug concentration (100 nmol/L) that does not affect cell viability after 24 hours. As shown in Fig. 3E, the drug treatment inhibited cell migration in both uveal melanoma cell lines.

### LNS8801 Treatment Induces Cell-cycle Arrest in G_2_–M by Disrupting Mitotic Spindle Function

**FIGURE 4:**
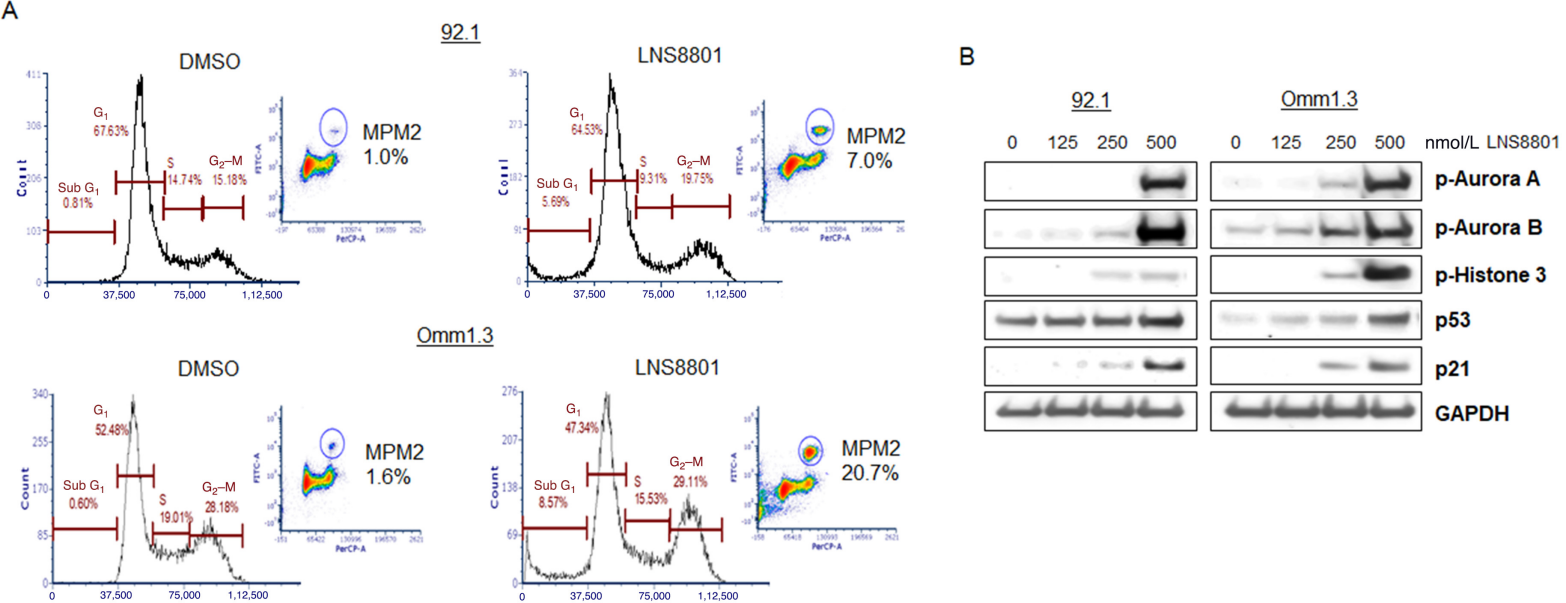
LNS8801 treatment induces cell-cycle arrest in G_2_–M-phase. **A,** Uveal melanoma cell lines were treated with DMSO or 500 nmol/L LNS8801 for 24 hours. Fixed cells were stained with propidium iodide and incubated with an antibody against the mitotic marker MPM-2 and analyzed for cell-cycle distribution by flow cytometry. Insets show mitotic cells positive for MPM-2. **B,** Uveal melanoma cells were treated with increasing doses of LNS8801 for 24 hours and analyzed by immunoblotting with antibodies for p-Aurora A, p-Aurora B, p-H3, p53, p21, and GAPDH.

**FIGURE 5:**
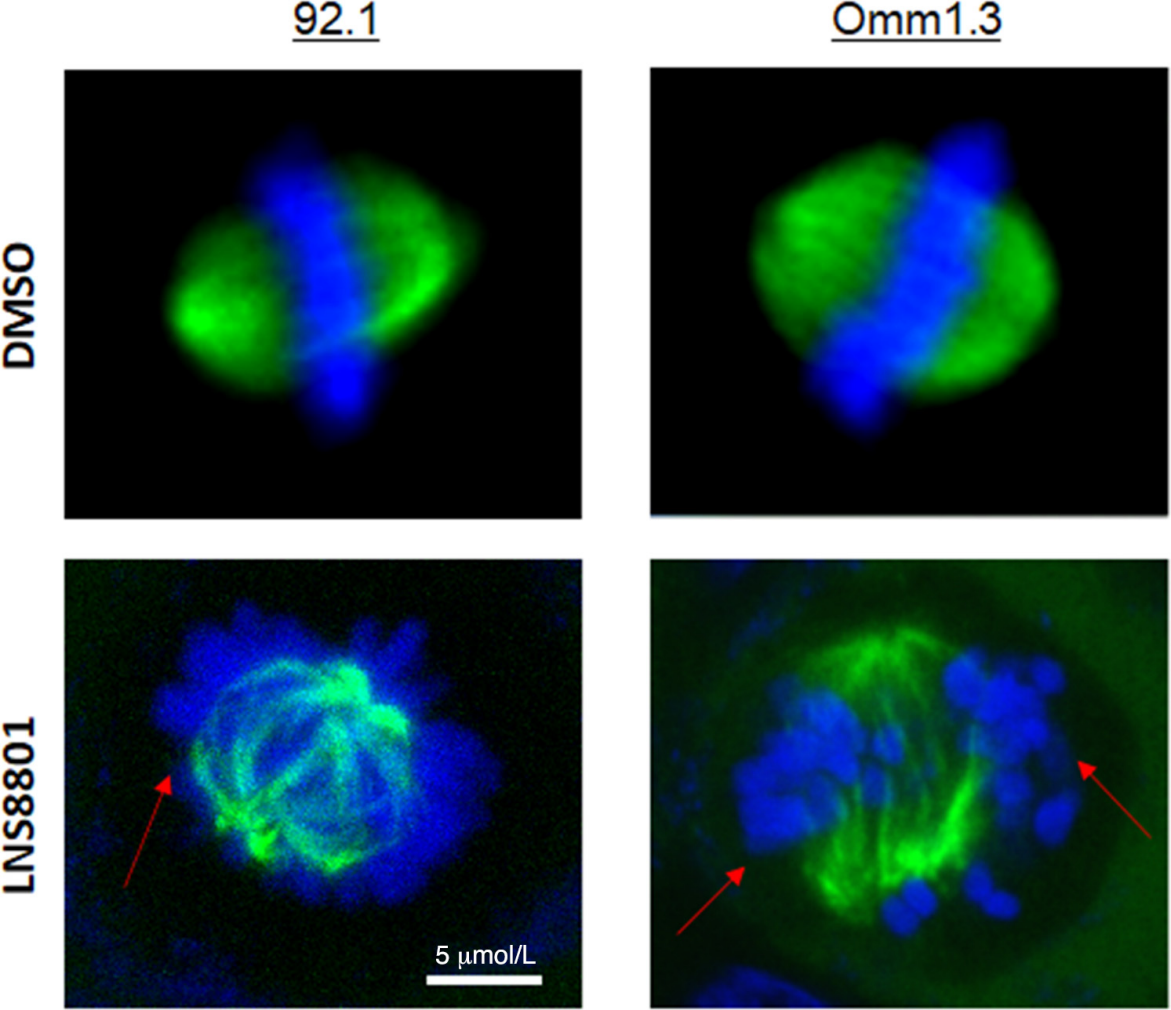
LNS8801 disrupts mitotic spindles. Representative images of 92.1 and Omm1.3 cells treated with DMSO or 500 nmol/L LNS8801 for 24 hours and immunostained with a FITC-tubulin antibody (green). DNA was stained with DAPI (blue). Images were taken on a confocal microscope. 60× magnification. Arrows indicate misaligned chromosomes.

To further explore the cellular mechanisms underlying LNS8801-induced inhibition of cell proliferation, we analyzed the cell-cycle profiles of 92.1 and Omm1.3 cells by flow cytometry. Cells exposed to 500 nmol/L LNS8801 for 24 hours showed an arrest in G_2_–M-phase of the cell cycle and concomitant increase of sub-G_1_ apoptotic cells (Fig. 4A). The mitotic marker MPM-2 also increased, from 1.0% to 7.0% and from 1.6% to 20.7% in 92.1 and Omm1.3, respectively (Fig. 4A). Consistent with the mitotic arrest of uveal melanoma cells, LNS8801 significantly induced the expression of mitotic proteins, such as the phosphorylated form of Aurora-A, Aurora-B, and Histone-3 in both cell lines, especially at 500 nmol/L dosage (Fig. 4B). Activation of GPER also induced p53 and p21, consistent with the results observed with GPER knockdown. To test whether the mitotic effects were mediated by GPER, the receptor was downregulated by siRNA in Omm1.3 cells (Supplementary Fig. S1A), and then treated with LNS8801 before mitotic index analysis. LNS8801 could still induce mitosis in cells with downregulated GPER (Supplementary Fig. S1B). Similar experiments were performed using the antagonist G-36. While G-36 pretreatment prevented the induction of p-CREB (Supplementary Fig. S1C), a known target of GPER, it was ineffective in blocking the induction of mitotic cells by LNS8801 (Supplementary Fig. S1D). These results suggest that the mitotic effects of LNS8801 are GPER independent. Effects on tubulin dynamics were reported with the preclinical agonist G-1 in ovarian, breast cancer, and glioma cells (19, 20, 28). Therefore, we evaluated the effect of LNS8801 on microtubules in uveal melanoma cells using a FITC-conjugated anti-tubulin antibody and DAPI to detect tubulin and DNA, respectively. Control mitotic cells showed normal spindles with aligned chromosomes (Fig. 5). In contrast, cells treated with LNS8801 for 24 hours formed defective mitotic spindles with misaligned chromosomes being outside of the spindle (Fig. 5).

### LNS8801 Inhibits Uveal Melanoma Growth *In Vivo*

**FIGURE 6:**
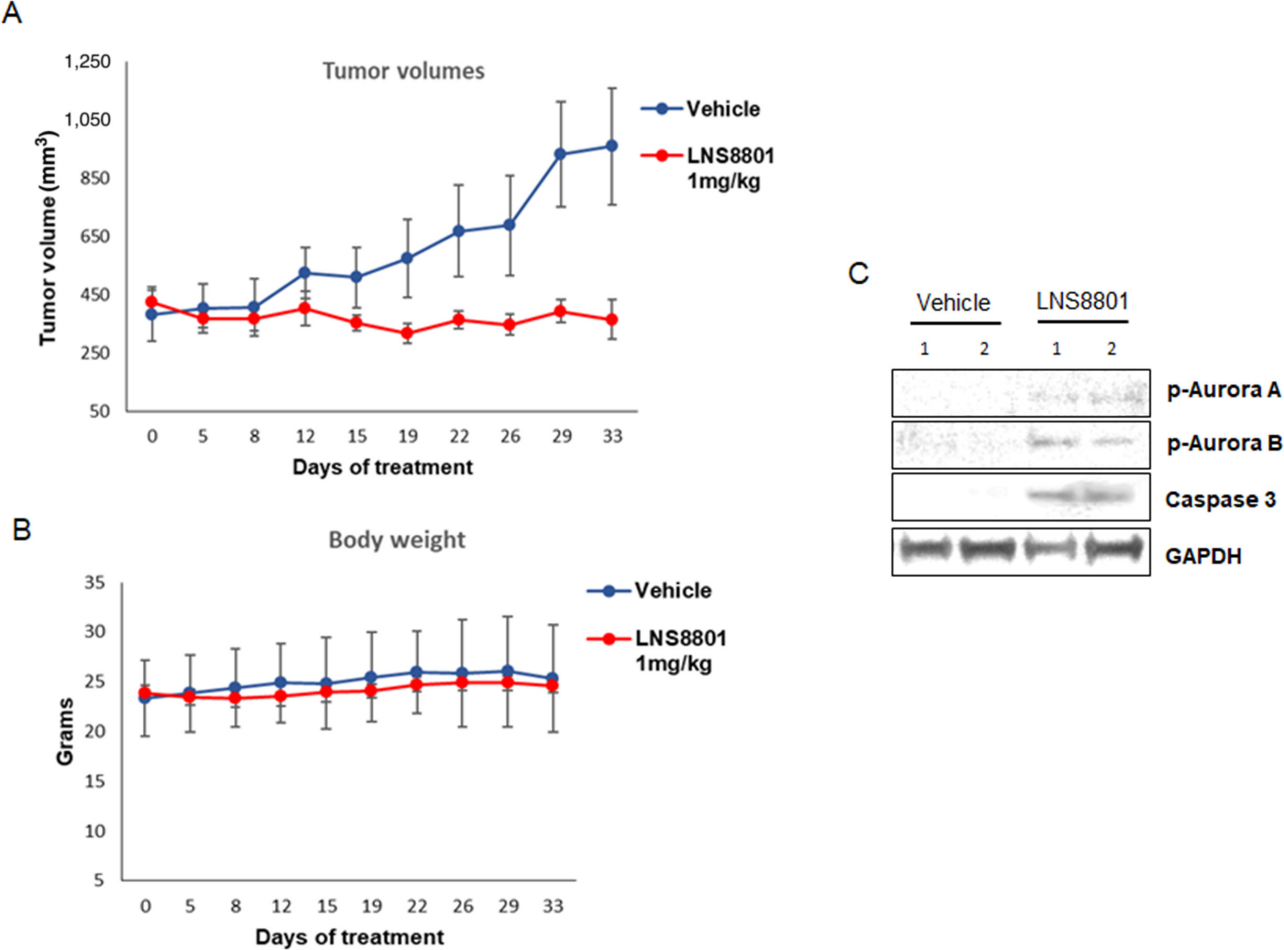
LNS8801 inhibits growth of uveal melanoma xenograft tumors *in vivo*. **A,** The mice were injected with 92.1 cells, and once the tumors developed, they were treated vehicle or 1 mg/kg LNS8801. Tumor volume was plotted against time ±SEM (*n* = 9). A longitudinal mixed effects model was used to assess the data and demonstrated a significant effect based on LNS8801 treatment versus vehicle and day of treatment, with *P* values <0.0015. **B,** Body weight of mice treated with vehicle or LNS8801 over time. Each point represents mean ±SEM. **C,** Tumors were excised and analyzed by immunoblotting probing for phosphorylated Aurora A and B, histone H3, caspase 3, and GAPDH as loading control.

The ability of LNS8801 to inhibit cancer cell growth was investigated in a xenograft mouse model of uveal melanoma. The mice were injected with 92.1 cells and when the tumors developed, the mice were treated with vehicle or 1 mg/kg/day LNS8801 orally for 5 weeks. As shown in Fig. 6A, LNS8801 induced significant suppression of tumor growth compared with vehicle over time without weight loss or other observations consistent with toxicity (Fig. 6B). Duplicate tumors collected after 21 days of treatment were analyzed by immunoblotting, and induction of p-Aurora A, p-Aurora B, total Aurora, and caspase 3 were observed in the treated tumors (Fig. 6C), confirming the effects of LNS8801 observed in uveal melanoma cell lines *in vitro*.

## Discussion

GPER is a membrane-bound estrogen receptor responsible for the rapid nongenomic effects of the estrogen response (29). Previous studies reported that gender plays an important role in cancer‐specific survival and drug response (30). It has been observed that women with uveal melanoma had better prognosis when compared with male patients (5, 7). While estrogen receptors are widely studied in cancer, including in uveal melanoma (31, 32), the role of GPER is still being defined. For instance, GPER may mediate the proliferative effects of estrogen in breast, endometrial, and in ovarian cancer cells in preclinical models (11), while other reports have shown a positive correlation of GPER expression with survival of patients with gastric and breast cancer (33, 34). In contrast, the treatment with the GPER agonist G-1 in preclinical studies has consistent antitumor activities in several cancers, including cutaneous melanoma (9), breast (24), ovarian (20, 35), pancreatic (17), and glioma (19).

Our study demonstrated potent antitumor effects of the clinical GPER agonist LNS8801 in uveal melanoma. We found that GPER is expressed in uveal melanoma cells and its downregulation stimulated cell growth. GPER knockdown was reported to increase growth of ovarian cancer cells (35), and there are studies demonstrating that GPER has tumor suppressor activities in breast cancer cells (23). Previous findings demonstrated that G-1 treatment induced p53 expression, and p53 knockdown partially reversed G-1–dependent anticancer effects in breast cancer cells (24), suggesting that p53 is a downstream mediator of GPER. We found that the receptor knockdown decreased basal p53 expression, a tumor suppressor protein that is rarely mutated in uveal melanoma (36), and it was upregulated by LNS8801 treatment. GPER-induced melanocytic differentiation was protective against cutaneous melanoma in mice (9). The treatment of uveal melanoma cells with the agonist LNS8801 also induced the expression of melanin and differentiation markers such as MITF, which directs transcription of melanocyte specific genes required for melanin synthesis, and the downstream effector TYR. Interestingly, LNS8801 further increased the expression of gp100, a melanoma antigen that has been effectively targeted by tebentafusp, a first-in-class bispecific fusion protein, recently approved by the FDA for the treatment of uveal melanoma (37). Tebentafusp is designed to target the melanoma-associated antigen gp100 through a high-affinity T-cell receptor binding domain and an anti-CD3 T-cell engaging domain, which redirects T cells to kill gp100-expressing tumor cells (38).

LNS8801 also induced an arrest in G_2_–M-phase of the cell cycle and a dramatic decrease in cell viability. Previous studies have reported direct effects of G-1 on tubulin polymerization, which prompted the formation of defective mitotic spindles and consequent induction of apoptosis in ovarian cancer and glioma cells (19, 20). Lv and colleagues reported that G-1 binds directly to the colchicine binding site on tubulin, inhibiting tubulin polymerization during breast cancer cell mitosis. These mitotic effects were not prevented by GPER antagonists (28), suggesting that the agonist has also GPER-independent effects. Our results on the tubulin dynamics in uveal melanoma cells are consistent with these previous observations (19, 39), because GPER downregulation and its pharmacologic blockade with the selective antagonist G-36 did not affect the ability of LNS8801 to induce cell-cycle arrest in mitosis. Schuler-Toprak and colleagues have identified 18 genes regulated by GPER knockdown and regulated in the opposite direction by G-1 treatment, clearly suggesting that these genes were GPER dependent (35). It appears, this family of GPER agonists elicits specific effects through GPER, and also microtubule-targeting effects that are independent of GPER (19). Our *in vivo* studies showed that LNS8801 suppressed growth of uveal melanoma xenograft in mice with no effects on mouse body weight or other signs of toxicity.

In a phase I dose-escalation study (NCT04130516) in patients with advanced cancer, LNS8801 was demonstrated to be safe and tolerable as both monotherapy and in combination with pembrolizumab (40, 41). It is plausible that the preclinical effects on mitotic spindle function may be occurring at concentrations different from the effective concentrations in humans and further studies will be required to definitively determine the contribution of GPER-dependent versus GPER-independent effects in patients. In the clinic, there are several lines of evidence suggesting that LNS8801 activity is linked to GPER signaling: (i) There are no signs of toxicity that would be characteristic of a microtubule inhibitor, even at doses and exposures above those required for clinical benefit (40); (ii) Progression-free survival across cancer types is highly correlated with expression of a functional/consensus version of GPER in patients (42); and (iii) A systemic, target engagement biomarker is highly correlated with progression free in patients across indications. The target engagement biomarker measures a rapid response to drug in the first few hours after dosing and follows the plasma exposure of LNS8801 (43).

In conclusion, our results provide evidence that LNS8801 has anticancer effects in uveal melanoma cells through GPER signaling and by targeting microtubule dynamics. While further studies will be needed to better define the activity of LNS8801, this agonist induced cell differentiation and suppressed tumor growth and may represent a promising treatment for uveal melanoma.

## Supplementary Material

Supplementary Figure S1 The mitotic effect of LNS8801 is GPER-independent
