# Supplementary material for: The GPER Agonist LNS8801 Induces Mitotic Arrest and Apoptosis in Uveal Melanoma Cells
Source: Cancer Res Commun. 2023 Apr 5;3(4):540–7. doi: 10.1158/2767-9764.CRC-22-0399 (PMC10075232; doi:10.1158/2767-9764.CRC-22-0399)
Supplement: Supplementary Figure S1 — The mitotic effect of LNS8801 is GPER-independent [file crc-22-0399-s01.pptx]

## Slide 1
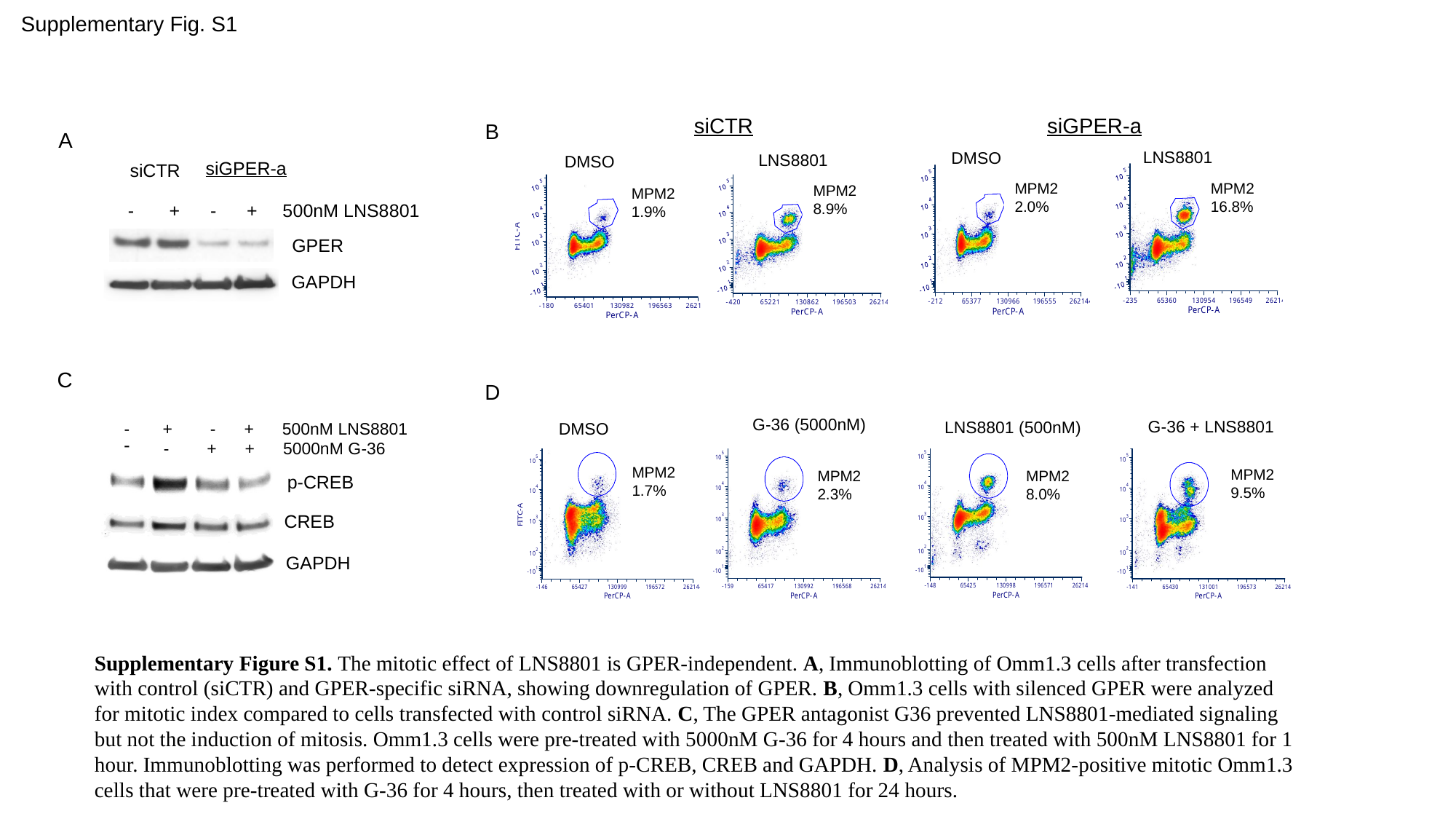

Supplementary Fig. S1
siCTR
siGPER-a
B
A
LNS8801
DMSO
LNS8801
DMSO
siGPER-a
siCTR
MPM2
16.8%
MPM2
2.0%
MPM2
1.9%
MPM2
8.9%
- + - + 500nM LNS8801
GPER
GAPDH
C
D
G-36 (5000nM)
G-36 + LNS8801
LNS8801 (500nM)
- + - + 500nM LNS8801
 - + + 5000nM G-36
DMSO
MPM2
1.7%
MPM2
2.3%
MPM2
8.0%
MPM2
9.5%
p-CREB
CREB
GAPDH
Supplementary Figure S1. The mitotic effect of LNS8801 is GPER-independent. A, Immunoblotting of Omm1.3 cells after transfection with control (siCTR) and GPER-specific siRNA, showing downregulation of GPER. B, Omm1.3 cells with silenced GPER were analyzed for mitotic index compared to cells transfected with control siRNA. C, The GPER antagonist G36 prevented LNS8801-mediated signaling but not the induction of mitosis. Omm1.3 cells were pre-treated with 5000nM G-36 for 4 hours and then treated with 500nM LNS8801 for 1 hour. Immunoblotting was performed to detect expression of p-CREB, CREB and GAPDH. D, Analysis of MPM2-positive mitotic Omm1.3 cells that were pre-treated with G-36 for 4 hours, then treated with or without LNS8801 for 24 hours.
